# Response from Authors to the Letter to the Editor

**DOI:** 10.1017/cjn.2020.225

**Published:** 2021-01

**Authors:** Juan Pablo Appendino, Steven Baker, Kristine M. Chapman, Tamara Dykstra, Tabrez Hussein, Michelle-Lee Jones, Michelle M. Mezei, Seyed M. Mirsattari, Marcus Ng, Joanne Nikkel, Vaso Obradovic, Cecile Phan, Lawrence Robinson, Angela Scott, José Téllez-Zenteno, Michelle Van Niekerk, Shannon Venance, Fraser Moore

**Affiliations:** Pediatric Neurology, Alberta Children’s Hospital, University of Calgary, Calgary, Alberta, Canada; Department of Medicine, Physical Medicine & Neurology, Peripheral Nerve Clinic, McMaster University, Hamilton, Ontario, Canada; Division of Neurology, Department of Medicine, University of British Columbia, St. Paul’s and Vancouver Hospital, Vancouver, British Columbia, Canada; EMG Clinic: Day Hospital, Riverview Health Centre, One Morley Avenue, Winnipeg, Manitoba, Canada; Diagnostic Neurophysiology, BC Children’s Hospital, Vancouver, British Columbia, Canada; Max Rady College of Medicine, Rady Faculty of Health Sciences, University of Manitoba, Winnipeg, Manitoba, Canada; Division of Neurology, Department of Medicine, Faculty of Medicine, University of British Columbia and Vancouver General Hospital, Vancouver, British Columbia, Canada; Departments of Clinical Neurological Sciences, Medical Imaging, Medical Biophysics and Psychology, Western University, London, Ontario, Canada; Max Rady College of Medicine, Rady Faculty of Health Sciences, University of Manitoba, Winnipeg, Manitoba, Canada; Neurodiagnostics Department, Health Sciences Center, Winnipeg, Manitoba, Canada; Neuromuscular Diseases Unit-VGH Diamond Health Care Center, Vancouver, British Columbia, Canada; Division of Neurology, Department of Medicine, Faculty of Medicine & Dentistry, University of Alberta, Edmonton, Alberta, Canada; Physical Medicine and Rehabilitation, University of Toronto, Toronto, Ontario, Canada; St. Joseph’s Health Care London, London, Ontario, Canada; Saskatchewan Epilepsy Program, Division of Neurology, Department of Medicine, Royal University Hospital, University of Saskatchewan, Saskatoon, Saskatchewan, Canada; Aviva Medical Specialists Clinic Inc., Barrie, Ontario, Canada; Department of Clinical Neurological Sciences, Schulich School of Medicine & Dentistry, Western University, London, Ontario, Canada; Department of Neurology and Neurosurgery, McGill University, Montréal, Quebec, Canada

We would like to thank Dr. Myers for taking the time to read our article^1^ and provide his comments.^2^ Dr. Myers raised a number of issues which we address here.

We did not include a detailed description of our methods in the article. This was intentional as we wanted it to be as brief and user-friendly as possible. We hope to guide practice in Canadian neurophysiology laboratories, but the article is not intended to replace local protocols in those places where they already exist.

To produce the guidelines, the authors were assigned to groups to review the published evidence up to the end of May, 2020, and provide their own experience regarding each of the topics included as headings in the published version. Each group wrote the first draft. These were combined into one document and this document was then circulated to all authors for feedback. Any issues or concerns that were identified with a topic were brought back to the attention of the original working group for that topic. After appropriate editing, the second draft was circulated. The remaining issues were discussed as a group until consensus was achieved.

Dr. Myers states that “the author list does not note anyone with expertise in public health or infectious disease. If the authors had reached out for input from other specialties such as these, the guidelines would carry more weight.”^2^ We did receive feedback from a colleague in Pediatric Infectious Diseases, who did not recommend any changes. As this colleague only reviewed the manuscript and did not participate in the consensus process, they were not included in the list of authors. We would like to strongly emphasize that this document is a collaboration between five Canadian organizations with expertise in Electrophysiology, including both physicians and technologists from a broad sample of Canadian Electrophysiology laboratories, all of whom work closely with the infection control departments of their institutions.

The third issue raised by Dr. Myers is whether hyperventilation should be performed or not. Dr. Myers states that we recommended that hyperventilation should be limited “only to *very high* yield clinical scenarios (*i.e.* childhood absence epilepsy)”.^2^ This is actually a misquote of our recommendation that hyperventilation should not be routinely performed, but “if justified for *high* diagnostic yield (*e.g.,* pediatric absence epilepsy)”,^1^ it could be performed. The differences are subtle but important. Dr. Myers is implying that we recommended that hyperventilation only be performed in exceptional circumstances where it is likely to be of very high yield. That was not our intention and not what was written. Dr. Myers discusses in detail the importance of including hyperventilation in the electrophysiological assessment of many patients in his practice. We completely agree with the idea that our recommendations need to be tailored to the needs of individual practices and laboratories. In the final section of our paper, we tried to emphasize the importance of local guidelines and this is another good example of that.

Dr. Myers also takes issue with our statement that “patients must wear surgical masks”^1^ when performing hyperventilation. He questions the references cited in support of this statement, provides three references of his own, and states that “this sort of misleading citation practice calls into question the reliability of the guidelines, particularly as the authors did not acknowledge other references providing very different guidance on the issue of hyperventilation, supported by accurate citations and rational argument.”^2^

We acknowledge that we were unable to provide strong published evidence in support of our recommendation at the time our article was submitted on June 12. As with many issues surrounding COVID-19, good published evidence did not exist at that time. We tried to provide readers with our consensus opinion as well as references for what little evidence did exist at that time.

From the three references cited by Dr. Myers, two were published after the submission of our article and one only just 2 days prior.^3^ We strongly disagree that they provide good evidence that hyperventilation should not be performed while wearing a mask. The article by Assenza et al.^4^ is a survey of Italian electrophysiology laboratories. A total of 121 laboratories performed hyperventilation with a mask and only 15 performed it without a mask. Despite this, the authors conclude that “hyperventilation should never be executed with mask.” They provide no references or evidence to support this statement.

The second article by Grippo et al,^5^ is a consensus statement, also from Italy. Five of the authors are the same as the Assenza article. The Grippo article states that “the execution of hyperventilation with the mask does not produce the physiological changes presumed to sustain an epileptiform EEG activation, thus it should never be performed.” They do not provide references or evidence to support their claim.

The third article is an editorial statement from a single hospital in Columbus, Ohio.^3^ This article was published online on June 10, 2020, but it is not accessible by the PubMed searching engine. The authors state that hyperventilation “should be performed only in confirmed COVID-19-negative patients. This can be completed with transient unmasking and with the parent (rather than the technician) holding a pinwheel for the child to blow. When COVID-19 testing prior to EEG is not available, hyperventilation should be performed only when absence seizures are suspected.” They do not discuss why they recommend transient unmasking.

**Figure 1:**
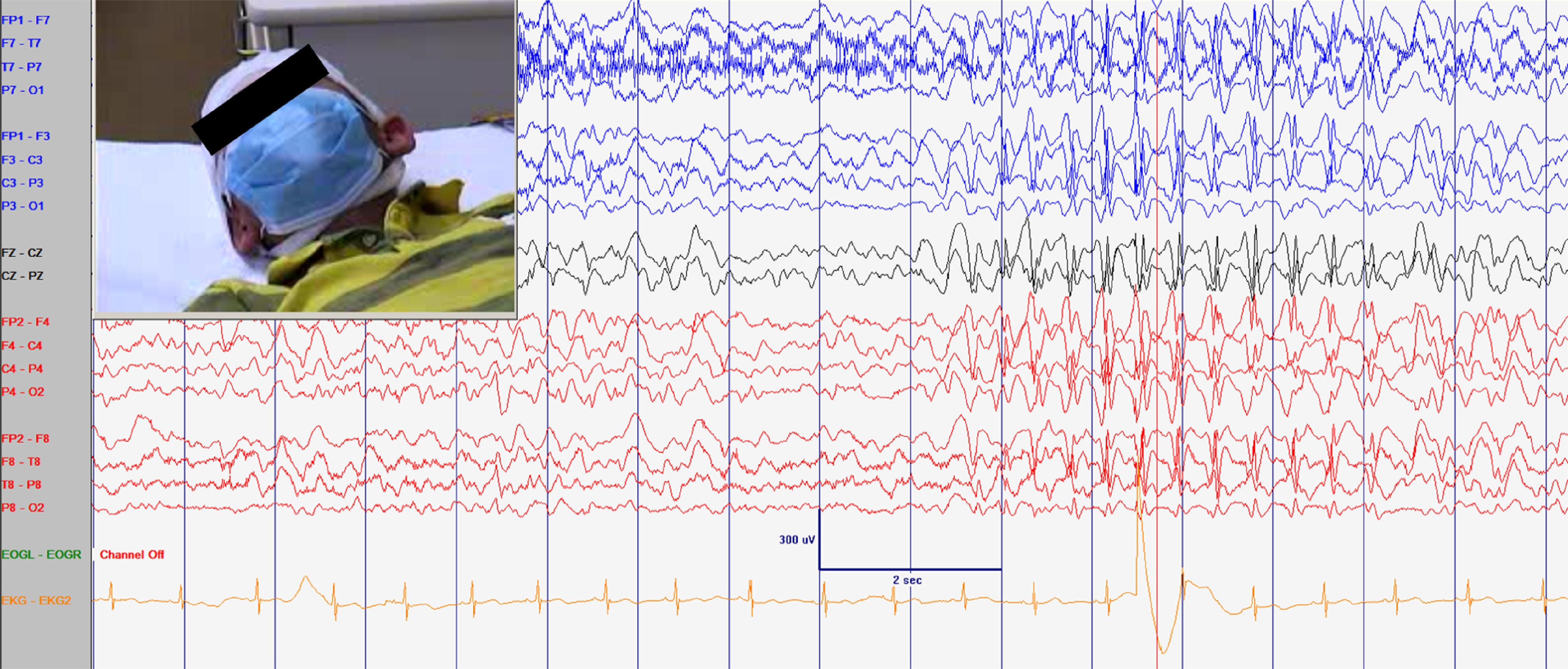
Patient hyperventilated (HV) for 3 min showing abnormalities usually observed during HV including slowing of the background and interictal discharges supporting that HV can be performed with a surgical mask.

In our expert opinion, and based on our experience, hyperventilation can be performed with a surgical mask in place (Figure 1). None of the references provided by Dr. Myers provide any evidence to refute this. Surgical masks are worn to prevent transmission of the COVID-19 virus via droplets from both symptomatic and asymptomatic individuals.^6^ These masks are not intended to provide an impermeable barrier that prevents gas exchange, nor do they provide a perfect seal. We feel it is unlikely that wearing a surgical mask would prevent a hyperventilating person from reducing their pCO2 level. Both the clinical and electrophysiological effects of hyperventilation can be easily monitored when a patient is in the electrophysiology lab. If for any reason these effects are not seen, and if hyperventilation is important to the evaluation of that individual patient, then consideration can always be given to repeating hyperventilation without a mask but with appropriate distancing.

Dr. Myers concluded by stating that he is “concerned about recommending changes to routine neurophysiology practices which would significantly decrease the diagnostic yield of tests when such changes are not supported by strong evidence, and there is no clear date or threshold as to when these changes would cease to be in effect”. We would like to emphasize that our expert recommendations were made in the extraordinary situation of a global pandemic. They were made with the dual intent of maintaining the highest standards of electrophysiology practice while at the same time limiting the spread of COVID-19.
